# Gut microbiota shape diurnal rhythms of amino acid metabolism in the mouse prefrontal cortex

**DOI:** 10.1016/j.molmet.2026.102319

**Published:** 2026-01-12

**Authors:** Gabriel S.S. Tofani, John F. Cryan

**Affiliations:** 1 APC Microbiome Ireland, Ireland; 2 Department of Anatomy & Neuroscience, University College Cork, Cork, Ireland

**Keywords:** Circadian rhythm, Gut microbiota, Gut-brain axis, Metabolomics

## Abstract

**Objectives:**

The gut microbiota plays a key role in maintaining brain health and homeostasis. Previous studies have demonstrated that metabolites in the brain respond to alterations in gut microbial composition. In this study we aimed to explore how depletion of the gut microbiota is associated with alterations in the diurnal rhythmicity of metabolites in the brain.

**Methods:**

We used antibiotic-induced microbial depletion in mice to examine the impact of the gut microbiota on the rhythmicity of metabolites in the prefrontal cortex. Metabolite profiles were assessed across multiple timepoints using untargeted metabolomics.

**Results:**

Microbial depletion was associated with alterations in the rhythmic profile of metabolites in the prefrontal cortex, with amino acids showing a robust inversion of their normal rhythm. These alterations were specific to the prefrontal cortex, with hippocampus and amygdala showing minimal changes. This altered gut microbial environment was associated with potential consequences for neurotransmitter production, including glutamate and serotonin.

**Conclusions:**

These findings provide further evidence that the gut microbiota shapes rhythmic diurnal processes in the brain. Future studies are warranted to investigate how such microbial effects influence actual neurotransmitter levels and behavioral phenotypes associated with the prefrontal cortex.

## Highlights

• Microbial depletion is associated with altered diurnal rhythms in the prefrontal cortex metabolome.
• Amino acid metabolism is sensitive to changes in gut microbial composition.
• Mice with depleted microbiota displayed an inversion of normal rhythm of amino acids in the prefrontal cortex.

Circadian rhythms are behavioral and physiological processes that occur over a 24-hour period [1]. Such rhythmicity is known to be key in maintaining health, regulating a myriad of biological functions that includes sleep, hormonal secretion, immune function, and metabolism [2]. The suprachiasmatic nucleus, located in the hypothalamus, is the central pacemaker which is responsible for synchronizing the rhythms between the different clocks throughout the body [3]. Moreover, circadian rhythmicity in other brain areas have been demonstrated to be essential for cognitive, emotional and social performance[[4], [5], [6]]. Although the main external input through which the central clock is modulated is light, recent findings have shown that gut-derived signals might also influence time-keeping processes in the brain [7,8].

Accumulating evidence has shown that gut microbiota can shape brain function and behavior [9]. Germ-free animals, born and raised in sterile conditions, exhibit alterations in anxiety-like behavior, stress responsivity, and social behavior among others [[10], [11], [12]]. Moreover, changes in the gut microbial environment, whether through antibiotics, diet, or disease, have also been shown to drive similar effects [[13], [14], [15], [16], [17]]. Although evidence that the gut microbiota can influence brain physiology is now plentiful, evidence that microbial composition can influence circadian processes in the brain remains scarce [18,19]. Specifically, the prefrontal cortex is sensitive to gut microbial signals [20,21], having robust clock processes [22]. Here we explore how microbial depletion affects the oscillation of metabolites in the brain to shed light on the role of the gut microbiota on circadian rhythmicity. Our data reveals that amino acid metabolism in the prefrontal cortex is especially vulnerable to changes in microbial composition.

**Figure 1:**
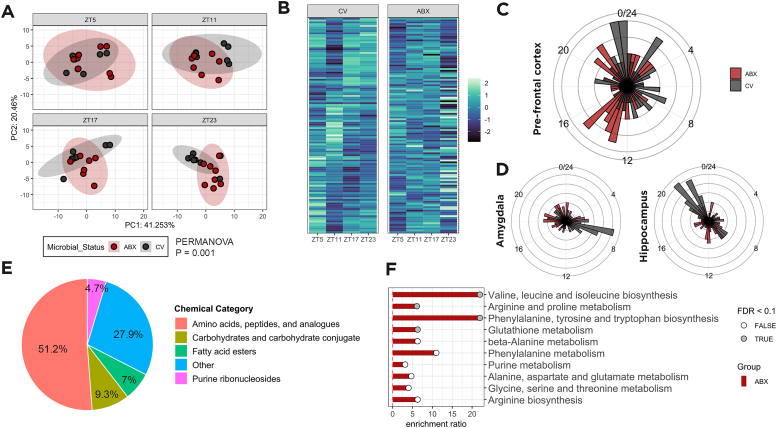
**Microbial depletion is associated with altered rhythmicity of the prefrontal cortex metabolome.** (A) Principal component analysis of the metabolome. Data analyzed with PERMANOVA (n = 7/8 per group/timepoint). (B) Heatmap of all metabolites present in the dataset. Acrophase plot of metabolites in the (C) prefrontal cortex, (D) amygdala, and hippocampus. (E) Classification of differently rhythmic metabolites between ABX and CV mice. Rhythmicity analysis was conducted by linear modeling to a Fourier-decomposed sine and cosine element for circadian rhythmicity followed by Tukey-adjusted post-hoc comparisons. (F) Enrichment plot of differently rhythmic metabolites. Data analyzed with Metaboanalyst.

To assess the diurnal dynamics of metabolites in the brain, we collected prefrontal cortex from antibiotic treated (ABX) and conventionally raised (CV) mice across four different timepoints: zeitgeber time (ZT) 5, 11, 17 and 23 and conducted untargeted metabolomics. Then, by employing a rhythmicity detection algorithm [23], we identified metabolites that are differently rhythmic between the two conditions. Principal component analysis revealed that the composition of the metabolome is significantly different between control and ABX, indicating the light/dark transitions as key timepoints (Figure 1A). Moreover, antibiotic induced microbial depletion led to a change in the overall rhythmic pattern of the metabolome (Figure 1B). These alterations in rhythmicity can be described as a shift of acrophase between the groups. Control animals had most metabolites peaking around ZT0 – just before the light onset – while ABX mice showed the majority of metabolites peaking around ZT14, demonstrating again that the changes are driven around the light/dark transitions (Figure 1C). This represented a robust shift of almost 12h in the distribution of acrophases. To determine if such alterations were exclusive to this brain region, we reanalyzed previously published data collected from the same animals [19] and showed that other brain regions did not show such patterns. In contrast with the prefrontal cortex, the amygdala and hippocampus both display an overall reduced amplitude in the rhythm of metabolites (Figure 1D). Those alterations in amplitude are associated with the loss of the marked peak in metabolites acrophases around ZT7 in the amygdala and ZT21 in the hippocampus. Together this data demonstrated that microbial manipulations are associated with region-specific shifts in the prefrontal cortex metabolome rhythmicity.

Next, we sought to understand if the described shift in metabolite rhythmicity was linked to a specific class of metabolites. To achieve this, we ran pairwise rhythm comparisons between the two groups to determine differential rhythmicity. A total of 49 out of 120 metabolites were differently rhythmic between controls and ABX mice. Here we show that among those altered metabolites mapped in MetaboAnalyst, over half of them were classified as amino acids or peptides (Figure 1E). Moreover, this was further confirmed as amino acids metabolism pathways were significantly enriched in this dataset (Figure 1F). These results demonstrated that the profound alterations observed in rhythmicity were attributed to changes in amino acid metabolism.

**Figure 2:**
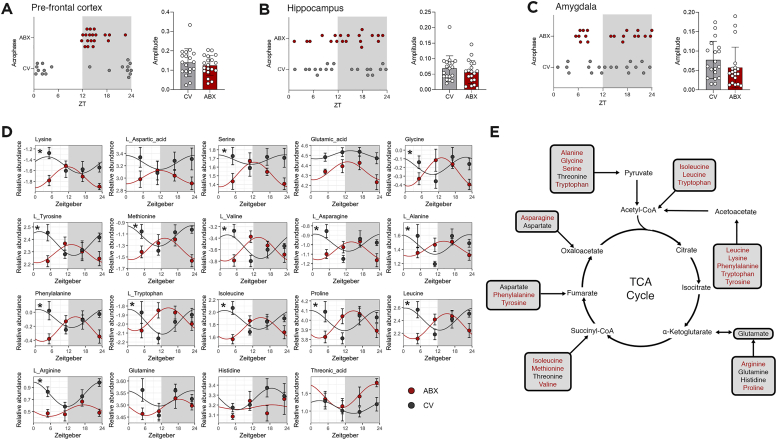
**Shift in amino acid metabolism rhythmicity drives changes in prefrontal cortex metabolome.** Distribution of acrophases and amplitude of amino acids in the (A) prefrotal cortex, (B) hippocampus, and (C) amygdala. (D) Abundance levels of common amino acids in the prefrontal cortex. Rhythmicity analysis was conducted by linear modeling to a Fourier-decomposed sine and cosine element for circadian rhythmicity followed by Tukey-adjusted post-hoc comparisons followed by Bonferroni correction. (E) TCA cycle schematic highlighting metabolites with altered rhythmicity in red. Data expressed as mean ± SEM. ∗*q* < 0.1.

Having defined that the changes driving altered rhythmicity in ABX mice were related to amino acid metabolism, we examined 19 out of the 20 common amino acids that were identified in our dataset. When observing the distribution of such metabolites, we can again observed a striking pattern in the prefrontal cortex. The selected amino acids peaked in the early dark phase in ABX mice, while in CV mice this happened in the transition from the dark to light phase (Figure 2A). Moreover, no changes in amplitude were detected. Once more, this pattern was demonstrated to be region specific, as the hippocampus and amygdala showed no difference in the distribution of acrophases of amino acids between CV and ABX mice (Figure 2B,C). This further demonstrates that the shift of rhythmicity in the metabolome is driven by alterations in amino acid metabolism.

**Figure S1:**
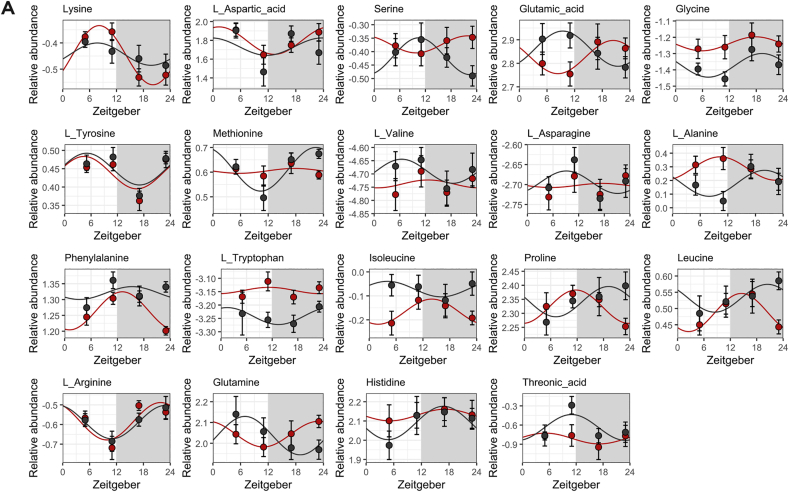
Abundance of amino acids across the day in the hippocampus. (A) Abundance levels of common amino acids in the hippocampus of CV and ABX mice. Rhythmicity analysis was conducted by linear modeling to a Fourier-decomposed sine and cosine element for circadian rhythmicity followed by Tukey-adjusted post-hoc comparisons. Data expressed as mean ± SEM.

**Figure S2:**
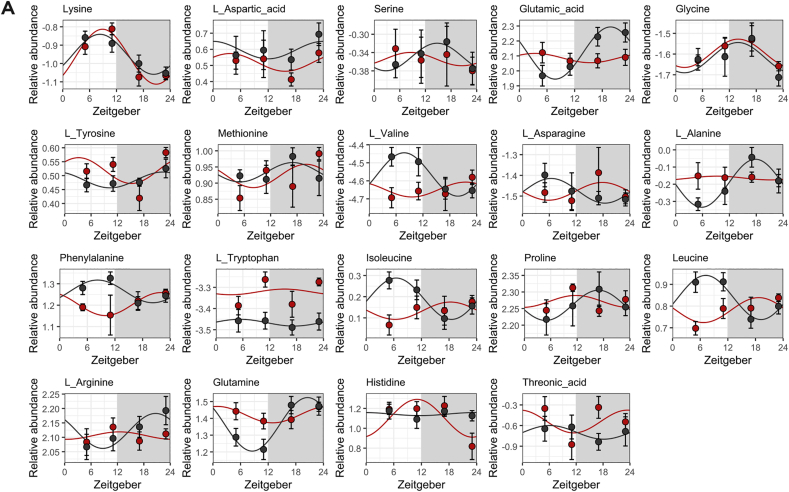
Abundance of amino acids across the day in the amygdala. (A) Abundance levels of common amino acids in the amygdala of CV and ABX mice. Rhythmicity analysis was conducted by linear modeling to a Fourier-decomposed sine and cosine element for circadian rhythmicity followed by Tukey-adjusted post-hoc comparisons. Data expressed as mean ± SEM.

Lastly, to understand the biological significance of this shift, we investigated each of these metabolites individually. Here we show that 14 out of the 19 amino acids were differently rhythmic between CV and ABX mice (Figure 2D). The shift in the peak of metabolites can be exemplified by amino acids such as valine, tryptophan, and tyrosine, among others. The same consistent pattern was not observed in the other two regions analyzed (Fig. S1 and S2). Amino acids are key to not only to protein synthesis but also serve as important components of the metabolism of different neurotransmitters such as glutamate, serotonin and dopamine [24]. As such, alterations in amino acid metabolism in the prefrontal cortex may have direct implications to behavioral outputs. Moreover, since these metabolites can be integrated into the tricarboxylic acid (TCA) cycle, disruptions to the rhythmicity of amino acids may also have implications for energy metabolism (Figure 2E).

In summary, this study provides evidence that the gut microbiota can influence diurnal rhythmicity of amino acid metabolism in the prefrontal cortex, and that its depletion leads to a marked reorganization of these rhythms. Among the altered pathways we observed in our dataset was the metabolism of branch chain amino acids (BCAAs), namely leucine, isoleucine and valine which are key to maintain metabolic health [25]. In the brain BCAAs are involved with different cellular functions such as protein synthesis and the generation of energy. This class of amino acids is able to cross the blood–brain barrier and once in the brain is involved in the production of glutamate, the main excitatory neurotransmitter [26,27]. This is especially relevant as glutamate is produced in the periphery, but it is not able to cross the blood–brain barrier. Moreover, glutathione metabolism was also enriched, further indicating that alterations in the microbiota composition can lead to altered glutamate metabolism in the brain. This further solidifies previous reports showing that the diurnal rhythmicity of glutamate metabolism in the brain is influenced by microbial manipulations [19]. Although in our dataset the rhythm of glutamate is not significantly altered between CV and ABX mice, the levels of this metabolite are consistently reduced throughout the day. Such alterations may help understand better the mechanisms through which the microbiota can alter cognition, social behavior, and stress responsivity, as these behaviors have been associated with alterations in glutamate metabolism in the prefrontal cortex [[28], [29], [30]].

Another interesting pathway that had its rhythmicity impacted by microbial depletions was tryptophan metabolism. Although the diurnal oscillation in tryptophan metabolism in the gut are well described [31], there is still a lack of information on how tryptophan metabolites changes across the day in the brain. Here we show that the rhythmicity of tryptophan in the brain is markedly disrupted, presenting an inversed pattern in ABX mice when compared to controls. As tryptophan is required to the production of serotonin, this finding may help explain the alterations in serotoninergic pathways observed in germ free mice [[32], [33], [34]].

Besides its role on the synthesis of neurotransmitters, amino acids in the prefrontal cortex have also been directly linked to behavioral outcomes. Rats that underwent chronic unpredictable mild stress showed altered amino acid metabolism in the prefrontal cortex, with some amino acids correlating with behavioral outputs [35]. Early-life stress impairs amino acid transport in the gut which is associated with synaptic dysfunction in the prefrontal cortex and is mediated by the gut microbiota [36]. Additionally, a rat model of attention-deficit/hyperactivity disorder is linked to changes in serine metabolism in the same region [37]. Lastly, kynurenine which is directly linked to tryptophan metabolism, is altered in the prefrontal cortex of depressed individuals [38]. Because amino acids in the prefrontal cortex are essential for maintaining normal behavior, our findings emphasize the influence of the gut microbiota and suggest new avenues to investigate how specific microbial compositions may causally contribute to behavioral changes.

A key finding is that these rather strong alterations in acrophase of amino acids are region specific, not being observed in the hippocampus and amygdala. This may be due to the brain region differences in blood–brain barrier permeability and expression of amino acid transporters [39,40]. Another important fact is that brain clock processes also vary widely between brain regions. Characterization of the diurnal transcriptome in primate brain revealed that the prefrontal cortex has significantly higher number of rhythmic transcripts when compared to the hippocampus and amygdala [22]. Another study has also demonstrated that the diurnal oscillations of metabolites in the prefrontal cortex are altered upon high-fat diet, and such changes are distinct than what was observed in the suprachiasmatic nucleus [41]. Together with our data, this indicates that different regions respond differently to peripheral signals, and that the robust rhythmic processes in the prefrontal cortex may be uniquely sensitive to changes in these signals.

Although the results presented here are valuable, this study has its own set of limitations. The lack of a dataset exploring these effects in germ-free mice prevents us from understanding if these effects are restricted to adulthood or could be influenced during development. Moreover, future studies should also include metabolomics data on germ-free animals under the same antibiotic cocktail, what would allow for clearer dissection of possible pharmacological effects of the antibiotics administered. Additionally, our study relies on untargeted metabolomics data, future studies should also perform targeted approaches to confirm the results here presented.

In our study we did not explore changes in food intake, which can directly affect amino acid metabolism. Although germ-free animals show increased food intake, their circadian eating patterns are preserved [42], what indicates that eating behavior itself may not underlie the significant effects on amino acids rhythmicity. Moreover, in our previous study we have demonstrated that the antibiotic cocktail used resulted in low-abundance rhythmic bacterial populations that peak in the light–dark transition [19]. This altered rhythmic microbiota coincides with the time of the strongest shifts in rhythmic amino acid metabolism. Together, this indicates that altered microbial composition and metabolic output may contribute to the alterations observed. However, further studies need to be conducted to test if it is the drastic reduction or shift in composition of gut microbes that are underlying the effects here presented, and what are the contributions of possible changes in food intake.

By demonstrating that microbial signals have both circadian and region specificity in their influence on brain metabolism, our findings pave the way for a deeper understanding of how gut microbes may influence brain function across the day. The results described here will provide a base for future studies investigating the mechanisms underlying the microbial modulation of mood disorders and cognition. Moreover, future studies will move towards more causal links between microbial-associated changes in rhythmicity and metabolism, integrating this with behavioral testing to reveal novel components of gut-brain communication.

Our research also highlights the importance of time-of-day in gut-brain axis and metabolism research. Although the importance of circadian rhythmicity in gut microbial composition and in metabolism are now recognized [43,44], a lot of studies still ignore this critical component. Since rhythmic processes have such a big impact both on metabolism and gut microbiota, accounting for time-of-day when investigating these topics is key to improving translatability and reproducibility. Moreover, this also underscores the importance of these rhythmic processes when designing microbiota-based therapies.

## 1 Methods

### 1.1 Animals

All experiments with animals were approved by the Animal Ethics Committee of University College Cork and Health Products Regulatory Authority (HPRA) under the project authorizations AE19130/P047 and carried out with accordance to the European Directive 2010/63/EU. Animals aged between 7 and 14 weeks were used during all experiments. C57/BL6 mice were acquired as breeding pairs from Taconic Biosciences, and subsequent generations were used. Mice were maintained in cages with 2–4 animals under a 12-hour light/dark cycle with *ad libitum* water and pelleted diet (Special Diet Services). Temperature was set to 21 ± 1 °C and humidity to 55%–60%.

### 1.2 Depletion of gut microbiota by antibiotic treatment

Mice received antibiotic treatment in *ad libitum* drinking water for a period of 14 days. The antibiotic cocktail consisted of ampicillin sodium salt (1 g/L), gentamicin sulfate (1 g/L), vancomycin hydrochloride (0.5 g/L) and imipenem (0.25 g/L) (Discovery Fine Chemicals). Water containing antibiotics was replaced every two days for the duration of the treatment and prepared freshly every time. Cages were randomly assigned to either vehicle or antibiotic treatment. Microbial depletion was verified by assessing bacterial load on cecal samples, results are in a previous publication [19].

### 1.3 Sample collection

Mice were transferred to a room adjacent to the dissection area and allowed to acclimate for 1.5 h prior to sacrifice. At each designated time point, animals were rapidly decapitated, and the brains were immediately removed. Brains were snap-frozen by immersion in isopentane cooled on dry ice and subsequently placed in a 1 mm brain matrix for sectioning. The prefrontal cortex was dissected using a scalpel within a cold chamber maintained at −20 °C to prevent thawing. Left and right hemispheres were pooled in the same tube for metabolomic analysis.

### 1.4 Metabolomics

Metabolomic profiling of the prefrontal cortex, hippocampus, and amygdala was performed by MS-Omics (Copenhagen, Denmark). Samples were extracted using a methanol–water (1:2) solution following bead-beating, ultrasonication, and centrifugation steps, with supernatants collected through Phree filters, dried under nitrogen, and reconstituted in 10% eluent B in eluent A prior to LC-MS analysis. A pooled quality control (QC) sample was prepared by combining aliquots from all samples and analyzed at regular intervals throughout the sequence. Semi-polar metabolites were analyzed using a Thermo Scientific Vanquish UPLC coupled to a Q Exactive HF mass spectrometer operated in both positive and negative electrospray ionization modes, following a slightly modified version of the method described by Catalin et al. (UPLC/MS Monitoring of Water-Soluble Vitamin Bs in Cell Culture Media in Minutes, Waters Application Note 2011, 720004042en). Data were processed using Compound Discoverer 3.2 and Skyline. Compound features were grouped and annotated based on accurate mass, isotopic pattern, and retention time information. Metabolites were assigned confidence levels according to MS-Omics’ internal standards: Level 1, confirmed by retention time, accurate mass (≤3 ppm), and MS/MS spectra; Level 2a, by retention time and accurate mass; Level 2b, by accurate mass and MS/MS spectra; and Level 3, by accurate mass and elemental composition alone. Further bioinformatic analysis was conducted on Levels 1 and 2a.

### 1.5 Bioinformatics and statistical analysis

**Figure S3:**
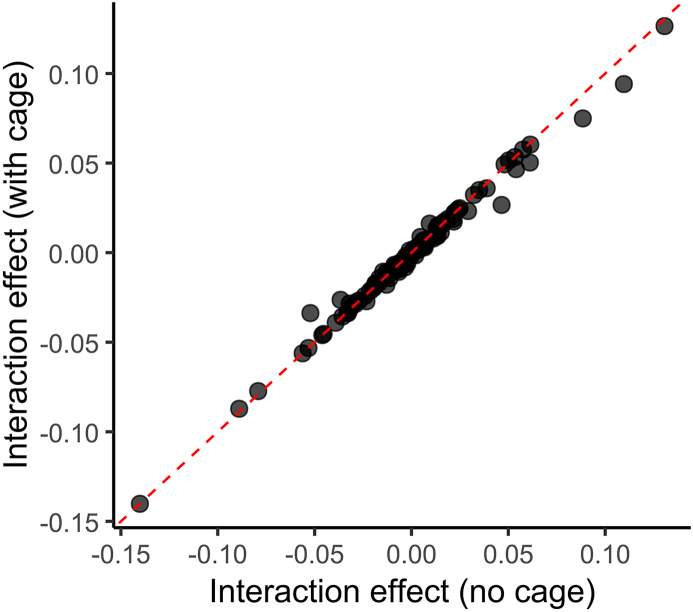
Cage effects have small impact on effect sizes. Scatter plot comparing the interaction effect estimates (β) for each metabolite calculated from linear models without cage (x-axis) and mixed-effects models including cage as a random effect (y-axis). Each point represents a single metabolite.

Biostatistics were undertaken in R (version 4.2.2) with the Rstudio GUI (version 2022.07.2 build 576). All data are represented as mean ± SEM. Data analysis was performed on centred log-ratio ratio (CLR) transformed values [45]. Principal component analysis was performed on CLR-transformed values. Zeroes were replaced using the ‘const’ approach described by Lubbe and colleagues [46]. The PERMANOVA implementation from the vegan library was used to find structural differences between metabolome profiles, the test was conducted on cage means to account for possible metabolome-wide cage effects. Rhythmicity analysis was conducted by fitting linear models for each CLR-transformed value [23]. Code used to fit these models can be found online at https://github.com/thomazbastiaanssen/kronos. To correct for multiple testing involved in the study, features were selected based on the Benjamini-Hochberg procedure with a q-value of 0.1 as a threshold. This procedure controls the False Discovery Rate (FDR) using sequential modified Bonferroni correction for multiple testing. Pairwise comparisons of rhythmicity were performed using a Tukey-adjusted post-hoc test. Differential rhythmicity was assessed via sine and cosine components of the time series, and Bonferroni correction was applied separately to both components. The lowest resulting q-value after correction was reported as the adjusted measure of differential rhythmicity. To evaluate whether cage influenced the effect of microbial status on metabolites over time, we fitted linear models for each metabolite with and without cage as a random effect. We extracted the estimated coefficient for the interaction term from each model to quantify the interaction effect and compared effect sizes between models to determine the impact of accounting for cage (Figure S3). Detailed statistical data and exact p-values are provided in Table S2. Custom R scripts and functions are available online at https://github.com/thomazbastiaanssen/Tjazi [47]. Enrichment analysis was conducted using the Metaboanalyst 6.0 online platform [48]. A FDR cut-off of 0.1 was used for pathway enrichment analysis.

## CRediT authorship contribution statement

**Gabriel S.S. Tofani:** Writing – review & editing, Writing – original draft, Visualization, Investigation, Formal analysis, Conceptualization. **John F. Cryan:** Writing – review & editing, Supervision, Funding acquisition, Conceptualization.

## Funding

The research was conducted in the APC Microbiome Ireland which is funded by 10.13039/501100001602Science Foundation Ireland (10.13039/501100001602SFI/12/RC/2273_P2). The research project was funded by the 10.13039/100019596Saks Kavanaugh Foundation.

## Declaration of competing interest

The authors declare the following financial interests/personal relationships which may be considered as potential competing interests:JFC has been an invited speaker at conferences organized by Bromotech and Nestle and has received research funding from Nutricia, Dupont/IFF, and Nestle. GTSS declares no competing interests.

## Data Availability

The data used on the manuscript is available at Table S1.

## Appendix A Supplementary data

Table S1 Raw prefrontal cortex (PFC) metabolomics data. Area values for each metabolite identified at Levels 1 and 2a. The first column indicates the treatment group and time point, and the second column contains the unique sample ID. Table S1

Table S2 Detailed statistical data. Table S2
